# The National Pension Fund

**Published:** 1891-01-03

**Authors:** 


### THE NATIONAL PENSION FUND.

It is surprising to those who are fighting in a battle what a different position they may occupy in the evening, from that which they held in the morning. The establishment of The National Pension Fund for Nurses has decidedly been something of a battle. At the opening of last year the hopeful atmosphere of victory was beginning to surround the advanced guard; at the close of the year the victory is complete and triumphant all along the line. In January the managers of the Fund asked for £40,000 wherewith to supplement the pensions of the deserving, and to relieve the necessities of the distressed; at the close of the year, not £40,000, but £50,000 are at the credit of those good objects, and more may be expected. The story of the Pension Fund, from the outset, reads like the pages of a novel. There was a time when both friends and foes alike stood wondering whether the ship would outride the storm, or would be swallowed up by the hungry waves. Let no dilettante critic say to himself that these figures of speech are stronger than the occasion demands:—there were waves that rose up against the Fund, and they were very hungry; and there is no doubt whatever that they would have swallowed it up with great satisfaction, and would have felt both their digestion and appetite improved by the process. But the fates were dead against them. The Pension Fund absolutely refused either to sink, or to be swallowed up. On the contrary, it breasted the waves like a good British ship until it came to the safe and beautiful harbour of Marlborough House. Who will grudge the founder and friends of the Fund a feeling of honest satisfaction that their work, after so many struggles and vicissitudes, should have been carried to such a happy termination? For it was a good work, a necessary work, and a far-seeing work; and of this no further proof was needed than that their Royal Highnesses the Prince and Princess of Wales should have given it their cordial sanction and approval. By way of making the National Pension Fund still more helpful and valuable to nurses, the Council have recently decided that it may be used either as an annuity office or a savings' bank. Nurses who enter for pensions, but who find that they are not able, for some unforeseen reason, to keep up their payments, may have all their premiums returned, with the addition of two and a-half per cent. interest per annum. The returned premiums will, of course, in justice to the nurses who continue to qualify for pensions, be subjected to a trifling reduction for working expenses. Furthermore, private nurses who fear to bind themselves to pay regularly a fixed sum every month, may pay into the Fund irregularly, their deposits bearing interest at 2½ per cent. By an automatic arrangement when the deposit amounts to £10, that sum will be turned into an annuity payment on the Returnable Scale. We shall publish full particulars of these important additional advantages, and meanwhile, any further information may be obtained from the Manager of the Fund, 8, King Street, E.C.

